# One-Year Follow-Up of Spa Treatment in Older Patients with Osteoarthritis: A Prospective, Single Group Study

**DOI:** 10.1155/2018/7492106

**Published:** 2018-07-02

**Authors:** Jolanta Zwolińska, Aneta Weres, Justyna Wyszyńska

**Affiliations:** Institute of Physiotherapy, Medical Faculty, University of Rzeszów, Rzeszów, Poland

## Abstract

**Introduction:**

Few studies evaluated the effects of spa therapy on pain perception and quality of life in older people with osteoarthritis. Therefore, the aim of the study was to evaluate the short- and long-term effects of spa therapy on quality of life and pain in patients aged 60 years and older with osteoarthritis.

**Materials and Methods:**

70 patients with generalized osteoarthritis were enrolled in the study. Spa treatment lasted 3 weeks (15 days of treatment) and was applied during a session lasting 120 to 150 minutes a day. All the patients benefited from kinesiotherapy, physical agent modalities, massage, peloid therapy, hydrotherapy with mineral waters, and crenotherapy. Visual Analogue Scale (VAS) for pain, the Laitinen scale, and WHOQOL-BREF questionnaire were used to assess the condition of the patients. The examinations were performed three times: at the beginning of the spa treatment, after three months, and one year after the first examinations.

**Results:**

Statistically significant improvements were observed in pain (VAS) between consecutive assessments (*p* <.001). Laitinen scale also reported beneficial, statistically significant changes in the level of pain (*p* <.001). The WHOQOL-BREF questionnaire reported a statistically significant improvement in the domain of social relations in 2-3 and 1-3 periods (*p *= .025 and* p *= .011, resp.). A significant improvement was recorded in the domain of environment between 2-3 and 1-3 periods (*p *<.001).

**Conclusion:**

Spa treatment reduced the level of pain in majority of the patients in short- and long-term follow-up and contributed to improving the quality of life in the domain of social relations and environment. To confirm the results of this study, there is a need for a randomized controlled trial comparing spa treatment with usual care in the older population with osteoarthritis.

**Trial Registration Number:**

This trial was retrospectively registered on 3 January 2018 with NCT03388801.

## 1. Introduction

Osteoarthritis is one of the most common conditions of the motor organ and the second cause of disability after cardiovascular conditions. Patients most frequently suffer from pain and stiffness of joints, which decrease the capacity of locomotion, self-care, and quality of life in the physical domain [1, 2]. In addition, the quality of life in the mental and social domain is diminished [3–5]. Problems in activities of daily living and decreased quality of life of patients often cause low mood or even depression [6, 7].

In 1994, World Health Organization established World Health Organization Quality of Life (WHOQOL) Group which defined the quality of life as individual's perceptions of own position in life in the context of their culture and value systems, together with their personal goals, standards, and concerns. It is influenced in a complex way by physical health, relationships with other people, and environment characteristics relevant for a subject [8–10]. In the case of chronic diseases, when medical goals frequently fail to be achieved, actions intended to improve the quality of life become significant [11].

The challenge of modern medicine is not only to prolong the life of a sick person, but also, above all, to improve the quality of life and to make it as close as possible to the condition before the disease. Therefore, currently, the interest in the research on the quality of life of people with various conditions is increasing [11, 12].

According to statistics in Poland, approximately 8 million people suffer from degenerative diseases [13]. Benefits related to sick leave, pensions, and early retirement due to disability generate significant economic losses [14]. Spa treatment is a valuable continuation of ambulatory or hospital therapy due to the complexity of applied methods. The central modality of spa treatment is balneotherapy, which is defined as the use of baths containing thermal mineral waters from natural springs at a temperature of at least 20°C and with a mineral content of at least 1 g/l. Spa therapy additionally employs physiotherapeutic interventions at a spa resort such as peloid therapy, crenotherapy, or inhalation of the mineral water [15].

Accordingly, patient-oriented treatments reduce pain and improve functions and functional capacity of patients. Going on a trip, changing lifestyle, resting from everyday hassles, and climate also have a positive impact on the patient's mental condition. Rehabilitation at the spa creates better conditions for physical therapies and triggers the return to social life [14].

In Europe, spa therapy is implemented in people with osteoarthritis. Out of 403,381 patients receiving spa treatment in the field of rheumatology in 2007 in France, nearly half of the patients had knee osteoarthritis. Spa therapies are funded by the social security system in France as well as in many other European countries [16].

Rehabilitation treatment at the spa resort creates better conditions for the use of physical therapies and accelerates the return to social life [17]. Despite numerous researches, high-quality scientific proofs for the effectiveness of spa treatments in osteoarthritis are scarce. The authors of systematic reviews conclude that the research undertaken so far is insufficient to unequivocally confirm the effectiveness of spa treatment and stresses the need for long-term follow-up studies [18, 19].

## 2. Purpose of the Paper

Therefore, the aim of the study was to evaluate the short- and long-term effects of spa treatment in people with generalized osteoarthritis on their quality of life and the perception of pain.

## 3. Materials and Methods

### 3.1. Study Design and Participants

The study was approved by the Bioethics Commission of the University of Rzeszow (no. 27/06/2016). In an interventional, prospective study design, we examined patients aged above 60 years (the WHO recognizes the age of 60 as the beginning of late older age) with the diagnosis of osteoarthritis who receive treatment in spa resorts in south-eastern Poland between April 2016 and July 2016. Out of all spa resorts in southeastern Poland, three spas (Horyniec Zdroj, Iwonicz Zdroj, and Rymanów) have been selected, which offer broad, comprehensive spa treatment. Patients were assessed by an experienced physiotherapist on the first day of the spa therapy (assessment 1), after three months (assessment 2), and one year (assessment 3) after the completion of the spa therapy. The study ended in August 2017. Written consent for participation was obtained from participants prior to the study. All subjects were informed about the possibility of dropping out at any stage of the study.

Inclusion criteria were as follows: age above 60 years, diagnosis of osteoarthritis, completion of 3 weeks of spa treatment, and patient's consent to participate in the study. All patients who qualified to the study did not receive spa therapy course at any time before. Exclusion criteria were the following: failure to complete 3-week spa treatment, significant random events during follow-up (such as the death of a family member, divorce, etc.), being diagnosed with other diseases during follow-up, other forms of therapy implemented during follow-up, and refusal to participate in 2nd and/or 3rd stage of study.

**Figure 1:**
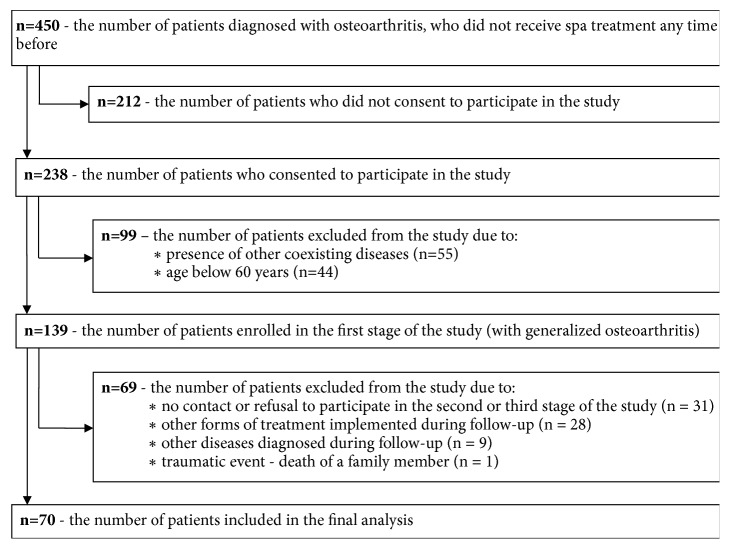
Flow diagram of study population.

A total of 450 patients with diagnosed osteoarthritis (based on medical records) were requested to participate in the study. 238 subjects agreed to participate in the study. Out of this group, a total of 99 people were excluded from the study due to the diagnosis of coexisting chronic diseases, which negatively affect the quality of life, and the age below 60 years. A total of 139 subjects were enrolled in the study and 70 patients were included in the final analysis (with generalized osteoarthritis). A detailed chart of the qualification to the study group is presented in Figure 1.

All patients who had been enrolled in the study were examined by an experienced physiotherapist on the first day of the spa therapy and asked to fill out the appropriate questionnaires. We ensured that questionnaires were properly completed under the supervision of researcher previously trained for their application. Second (assessment 2) and third (assessment 3) stages of the study were performed after three months and one year after the completion of spa treatment, respectively (participants were interviewed by telephone survey).

### 3.2. Interventions

Inpatient spa treatment was applied during a session lasting 120 to 150 minutes a day. Spa treatment lasted 3 weeks, including treatments from Monday to Friday (15 days of treatment). As a part of comprehensive spa treatment, all the patients benefited from morning workout for 15 minutes, water therapy exercise for 30 minutes, transcutaneous electrical nerve stimulation (TENS) for 20 minutes, low level laser therapy (LLLT) with 8 J/point and 400 mW, infrared irradiation for 15 minutes, classic massage for 20 minutes, peloid therapy for 20 minutes, hydrotherapy with mineral waters for 20 minutes daily, and crenotherapy (hydrogen sulphide and inorganic sulfides water, chloride-hydrogen-carbonate-sodium, iodide, and acidulous water). Specification of hydrogen sulphide and inorganic sulfides water is Na^+^, K^+^, Li^+^, NH_4_^+^, Ca^2+^, Mg^2+^, Fe^2+^, F^−^, Cl^−^, SO_4_^2−^, HCO_3_^−^. Mineralization of this water is 710-820 mg/dm^3^. Hydrogen sulfide level is 34,7-49,6 mg/dm^3^. Specification of chloride-hydrogen-carbonate-sodium, iodide, and acidulous water is Na^+^, K^+^, Li^+^, NH_4_^+^, Ca^2+^, Mg^2+^, Fe^2+^, Sr^2+^, Ba^2+^, F^−^, Cl^−^, HCO_3_^−^, Br^−^, J^−^. Mineralization of this water is 10806,3974 mg/dm^3^.

The specification of the peloids used is as follows: (a) solid content (non degrate) ca. 1%, (b) Von Post Humification Scale: H6 or H7, (c) pH: 6,06-6,26, (d) water content: 89,6-90,3%, (e) organic substances: 96,48-95,07% of dry weight, (f) inorganic substances: 3,52-4,90% of dry weight, and (g) silica: 0,03-0,05% of dry weight.

### 3.3. Outcome Measures

Pain intensity in the past 7 days was measured using Visual Analogue Scale (VAS), where 0 indicates no pain or best and 10 indicates the most intense pain imaginable or worst [20].

Assessment of pain intensity according to the Modified Laitinen Pain Questionnaire was also performed. The Modified Laitinen Pain Questionnaire contains questions about pain intensity, frequency of pain, frequency of using painkillers, and mobility. A total score in these four domains ranges from 0 to 16, with lower score indicating a better subject's condition [20].

The Polish version of the World Health Organization Quality of Life WHOQOL-BREF questionnaire (shortened version based on WHOQOL-100) was used to evaluate quality of life. The questionnaire assesses the quality of life, taking into account four domains of life: physical, psychological, social, and environment. The questionnaire consists of 26 questions to which the patient responds. Answers are awarded a score in a five-point scale (1-5). Answer scores are calculated according to the WHOQOL-BREF algorithm in the range of 0-100 points. Higher score corresponds to higher quality of life [21].

### 3.4. Data Analysis

Three study periods were considered: between the first and the second assessment (1-2), between the second and the third (2-3) assessment, and between the first and the third assessment (1-3). Statistical analysis of the results was developed using STATISTICA 10.1. Statistical analysis of collected data used order statistics and Wilcoxon's test, adopting the significance level at* p* < .05.

## 4. Results

The results of 70 patients (43 women and 27 men) with generalized osteoarthritis were analyzed in the study. The age of the respondents ranged from 60 to 80 years; most of the respondents were aged between 65 and 75 years. The detailed characteristics of the subjects are presented in Table 1.

Reduction in the pain level measured in VAS scale was statistically significant in three assessment periods (*p* < .001). The use of Modified Laitinen Pain Questionnaire enabled the demonstration of short- and long-term effects of spa treatment, with the long-term effect of treatment being clearer than the short-term effect. The observed changes were statistically significant in three assessment periods (*p* < .001) (Table 2).

The assessments of quality of life in the physical domain were similar in the beginning and at the end of the spa treatment. The quality of life in this domain was rated at approx. 60 pts, which can be considered as an average score. There have been no changes in the quality of life after spa treatment.

Also in the psychological domain, the impact of spa treatment on quality of life has not been confirmed. Long-term follow-up values were higher by approximately 2 pts, but the change recorded in the period (1-3) was not statistically significant (*p* = .164).

At the end of the treatment, statistically significant improvement in social relations was observed. The quality of life in this domain was by 1.4 pts higher after treatment and up to 5.0 pts a year later than the baseline. Changes in the periods 2-3 and 1-3 were statistically significant (*p* = .025 and* p* = .011, resp.).

The most satisfactory results of spa treatment expressed by improvement of quality of life were noted in the domain of environment. Immediately after the treatment, the quality of life in this domain was about 2 pts higher on average compared to the first assessment, with an average improvement of 8.1 pts per year. Changes in the periods 2-3 and 1-3 were very statistically significant (*p* < .001) (Table 3).

## 5. Discussion

To our knowledge, this study is the first to examine the long-term effects of spa treatment on quality of life and pain perception in older people with generalized osteoarthritis. Data from our study indicated a beneficial, long-term effect of spa therapy on pain and quality of life in social relationships and environmental domains in patients with osteoarthritis.

The analysis of contemporary literature shows that the quality of life in the context of health and disease is one of the most important areas of research in modern medicine. This subject still raises great interest, and despite numerous clinical studies, it has not been fully explored [22, 23]. Comprehensive treatment includes a variety of balneotherapeutic and kinesitherapy treatments and is linked to changes in diet, professional and social activity, and family life. All these elements combined with the possibility of daily recreation in the spa resort provide effective rest. The environmental conditions in the spa, lack of stress, and rest from professional and domestic duties reduce increased muscle tension as well as pain and promote the biological renewal of the patient [23, 24].

Coccheri et al. demonstrated lower rates and lower costs of health services in the patients with cardiovascular, respiratory, digestive, urinary, and reproductive tract diseases [25]. Dandinoglu et al. found that balneotherapy improves gastrointestinal motility and reduces constipation in the middle-aged and the elderly individuals [26]. Nitera-Kowalik et al. emphasized that spa treatment plays an important role in the process of positive aging free from disability and functional disorders. Patients in the age group of 50 to 70 years, in whom the above effects of spa treatment can significantly influence the level of quality of life, are predominant in our studies [27].

In patients with degenerative changes beyond the thermal and mechanical effects of therapeutic waters, their constituents are very important, for example, sulfur, which has a specific effect on articular cartilage [28]. Benedetti et al. evaluated the influence of spa therapy based on sulfur baths on biomarkers of oxidation, inflammation, and decomposition of articular cartilage. According to the authors, sulfur baths used in patients with osteoarthritis are a valuable complement to pharmacotherapy. In addition, the combination of sulfur baths and peloid treatment is recommended for preservation of therapeutic effects [29]. Also, according to Bellometti et al., regular peloid treatment with mineral baths reduces the use of painkillers and physiotherapy [23]. In Fioravanti et al.'s work, the effects of sulphate-bicarbonate-calcium mineral baths on the reduction of symptoms and changes in the quality of life of patients with osteoarthritis were also assessed. After 15 days of therapy in the spa, pain reduction and functional improvement were observed. There was good tolerance of balneotherapy and only a few and transient side effects. According to the authors, the implemented treatment is a valuable addition to pharmacotherapy and an alternative for the patients who do not tolerate pharmacological treatment [30]. In another study, Fioravanti et al. considered the long-term effects of mud-bath therapy on quality of life and pain perception in people with bilateral knee osteoarthritis. Authors observed a statistically significant improvement in pain perception and physical function at the end of the treatment and also at 3 months of follow-up. The control group did not show significant differences between baseline time and all other times [31].

Karagülle et al. have shown that spa treatment reduces pain and improves physical fitness and general well-being in older people with osteoarthritis [32]. In another study, authors found significant improvements in pain and function scores after outpatient balneological treatment consisting of hydrotherapy and peloid therapy in elderly patients with generalized, knee, lumbar, cervical, and hand osteoarthritis [33]. Also Erol et al. studied the effectiveness of spa treatment implemented in patients with generalized osteoarthritis. Improved functional status, reduced pain, and improved quality of life were observed. According to the authors, the spa treatment may improve the clinical status of patients with osteoarthritis and seems to be well tolerated [34]. In our study, the treatment used in the patients was similar to the treatment implemented in the above studies. The patients also did not report adverse symptoms and side effects. It should be emphasized that some patients reported transient worsening of symptoms during the first week of stay in the spa, which could be explained by intensive (strongly stimulating) effect of such treatment on the body. Gaál et al. assessed the effects of 30-minute daily baths in mineral water on chronic musculoskeletal pain, functional capacity, and quality of life in elderly patients with osteoarthritis of the knee or with chronic low back pain. Compared to baseline, all evaluated parameters were significantly improved. Moreover, the favorable effect was prolonged for 3 months after treatment [35].

Nitera-Kowalik et al. demonstrated that 21-day rehabilitation performed in the spa reduces the feeling of pain and improves functional capacity in everyday life [27]. In contrast, the results of Vaht et al. in 296 patients confirmed the efficacy of 6 and 12 days of spa treatment [36]. The patients who were included in our study used a three-week spa treatment. In our conditions (in Poland), this is the most commonly implemented form of spa treatment financed by the National Health Foundation.

Psychotherapy is an important element of comprehensive spa therapy. It contributes to the improvement of the emotional and mental condition and to the effectiveness of somatic treatment and facilitates coping with difficult situations and improves social relations [6]. This is confirmed by the results of our study, where there has also been an improvement in social relationships and functioning in the environment in the long-term perspective.

Benedetti et al. recommend cyclic repetition of spa therapy [29]. Fioravanti et al. showed that the balneotherapy effects lasted at least 12 weeks [30]. In Erol et al.'s study, the effects of spa treatment lasted up to eight months [34]. Samborski and Ponikowska, on the other hand, stated that the clinical improvement achieved after health resort therapy could last up to a year [28]. In our studies, it was confirmed that the effects of spa treatment were maintained one year after the end of the treatment.

Quality of life is the most important indicator of the effectiveness of medical and social care in a given society. Maintaining this indicator at the highest level requires access to spa treatment. The trip to the spa is not only a method of overcoming pain and functional limitations for the older people, but also an escape from fear of disease and its progress, loneliness, or social isolation. An indispensable condition for maintaining high availability for spa treatment is to document its effectiveness in clinical studies according to the requirements of Evidence Based Medicine.

*Limitation*. The limitations of our paper are a small number of respondents who participated in three consecutive studies and lack of a control group. The limited number of respondents is related to such factors as difficulty in contact after a longer period of time or reluctance to participate in subsequent surveys. The absence of the control group (untreated) is due to the fact that patients with osteoarthritis cyclically take part in various forms of treatment. It should also be stressed that many factors make it difficult to make an objective assessment of long-term effects (even unobserved events in everyday life may affect the mood, attitude, and responses of the respondents). To confirm the results of this study, randomized controlled trials comparing spa treatment with usual care in the older population with osteoarthritis are required as well as additional studies to clarify the mechanisms of action and the effects of the application of spa therapy.

## 6. Conclusions

The implementation of spa treatment in people with osteoarthritis allows for short- and long-term analgesic effects. Spa treatment allows improving the quality of life in terms of social relations and functioning in own environment in the long-term perspective.

## Figures and Tables

**Table 1:** Detailed characteristics of the study group.

**Variable**	**n**	%
**Sex**		

Female	43	61.4
Male	27	38.6

**Place of residence**		

City	42	60.0
Village	28	40.0

** Spa resort**		

Horyniec Zdrój	32	45.7
Iwonicz Zdrój	27	38.6
Rymanów Zdrój	11	15.7

**Type of work performed**		

White-collar work	36	51.4
Blue-collar work	30	42.9
Mixed	4	5.7

**BMI classification**		

Underweight	1	1.4
Normal	19	27.1
Overweight	30	42.9
Obesity	20	28.6

**Table 2:** Pain intensity in VAS and the Modified Laitinen Pain Questionnaire.

Variable	$\overset{-}{x}$	**Me**	***s***	**min**	**max**
**Pain intensity (VAS)**					

Assessment 1	5.5	5.5	1.9	0	9
Assessment 2	4.0	4	2.3	0	10
Assessment 3	2.5	2	2.1	0	7
Assessment 2 versus assessment 1 **(*p* < .001)**	-1.4	-1	2.2	-8	5
Assessment 3 versus assessment 2 **(*p* < .001)**	-1.5	-1	2.1	-7	2
Assessment 3 versus assessment 1 **(*p* < .001)**	-3.0	-3	2.2	-9	2

**Pain intensity (Modified Laitinen Pain Questionnaire)**					

Assessment 1	5.9	6	2.5	0	12
Assessment 2	4.2	4	2.3	0	9
Assessment 3	2.8	3	2.1	0	9
Assessment 2 versus assessment 1** (*p* < .001)**	-1.7	-2	2.2	-6	3
Assessment 3 versus assessment 2 **(*p* < .001)**	-1.4	-1	1.9	-6	3
Assessment 3 versus assessment 1 **(*p* < .001)**	-3.1	-3	2.4	-8	2

*p* is the probability value calculated using the Wilcoxon test.

**Table 3:** Quality of life of the study group.

**Quality of life domains**	$\overset{-}{x}$	**Me**	***S***	**Min**	**Max**
**Physical**					

Assessment 1	59.5	60.7	10.9	35.7	85.7
Assessment 2	59.0	60.7	10.0	28.6	85.7
Assessment 3	60.9	60.7	7.6	42.9	78.6
Assessment 2 versus assessment 1 (*p* = .573)	-0.5	0.0	10.6	-21.4	25.0
Assessment 3 versus assessment 2 (*p* = .138)	1.9	0.0	9.2	-25.0	32.1
Assessment 3 versus assessment 1 (*p* = .297)	1.4	0.0	10.3	-21.4	28.6

**Psychological**					

Assessment 1	67.1	66.7	12.4	41.7	91.7
Assessment 2	67.5	68.8	11.3	37.5	87.5
Assessment 3	69.0	70.8	9.7	41.7	83.3
Assessment 2 versus assessment 1 (*p* = .548)	0.4	0.0	9.0	-20.8	16.7
Assessment 3 versus assessment 2 (*p* = .232)	1.5	0.0	11.2	-33.3	41.7
Assessment 3 versus assessment 1 (*p* = .164)	2.0	4.2	13.0	-37.5	33.3

**Social relationships**					

Assessment 1	76.1	75.0	17.1	41.7	100.0
Assessment 2	77.5	83.3	16.4	41.7	100.0
Assessment 3	81.1	83.3	14.7	25.0	100.0
Assessment 2 versus assessment 1 (*p* = .455)	1.4	0.0	12.3	-33.3	33.3
Assessment 3 versus assessment 2 **(*p* = .025)**	3.6	0.0	11.1	-16.7	33.3
Assessment 3 versus assessment 1 **(*p* = .011)**	5.0	0.0	14.3	-33.3	41.7

**Environment**					

Assessment 1	71.3	71.9	13.5	37.5	100.0
Assessment 2	73.3	73.4	12.9	31.3	100.0
Assessment	79.4	81.3	9.8	40.6	96.9
Assessment 2 versus assessment 1 (*p* = .186)	2.0	0.0	12.1	-25.0	31.3
Assessment 3 versus assessment 2 **(*p* < .001)**	6.2	6.3	10.0	-18.8	37.5
Assessment 3 versus assessment 1 **(*p* < .001)**	8.1	6.3	12.8	-15.6	50.0

*p* is the probability value calculated using the Wilcoxon test.

## Data Availability

The datasets used and/or analyzed during the current study are available from the corresponding author upon reasonable request.
